# The Great Lockdown in the Wake of COVID-19 and Its Implications: Lessons for Low and Middle-Income Countries

**DOI:** 10.3390/ijerph19010610

**Published:** 2022-01-05

**Authors:** Sigamani Panneer, Komali Kantamaneni, Vigneshwaran Subbiah Akkayasamy, A. Xavier Susairaj, Prasant Kumar Panda, Sanghmitra Sheel Acharya, Louis Rice, Champika Liyanage, Robert Ramesh Babu Pushparaj

**Affiliations:** 1 Coordinator—Centre for Happiness, Department of Social Work, School of Social Sciences & Humanities, Central University of Tamil Nadu, Thiruvarur 610005, India; 2 Faculty of Science and Technology, University of Central Lancashire, Preston PR1 2HE, UK; 3 Department of Social Work, School of Social Sciences & Humanities, Central University of Tamil Nadu, Thiruvarur 610005, India; vickyfrommadurai@gmail.com (V.S.A.); robertrb19@students.cutn.ac.in (R.R.B.P.); 4 Research Department of Economics, Sacred Heart College, Tirupattur 635601, India; floraxavier@rediffmail.com; 5 Department of Economics, School of Management, Pondicherry University, Puducherry 605014, India; pkp.pondyedu@gmail.com; 6 Centre of Social Medicine and Community Health, School of Social Sciences, Jawaharlal Nehru University, New Delhi 110067, India; sanghmitra.acharya@gmail.com; 7 Centre for Architecture and Built Environment Research, University of the West of England, Bristol BS16 1QY, UK; Louis.Rice@uwe.ac.uk; 8 School of Engineering, Faculty of Science and Technology, University of Central Lancashire, Preston PR1 2HE, UK; clliyanage@uclan.ac.uk

**Keywords:** COVID-19 pandemic, the great lockdown, economic slowdown, health impact, migrants, informal sector, public policy, low- and middle-income countries

## Abstract

Concern for public health has been growing with the increasing volume of cases of COVID-19 in India. To combat this pandemic, India has implemented nationwide lockdowns, and unlocking phases continue with certain restrictions in different parts of the country. The lockdown has required people to adopt social-distance measures to minimize contacts in order to reduce the risks of additional infection. Nevertheless, the lockdown has already impacted economic activities and other dimensions of the health of individuals and society. Although many countries have helped their people through advanced welfare protection networks and numerous support aids, several emerging economies face specific difficulties to adapt to the pandemic due to vulnerable communities and scarce resources. However, certain lower-income countries need more rigorous analysis to implement more effective strategies to combat COVID-19. Accordingly, the current systematic review addresses the impacts of the COVID-19 pandemic and lockdowns in India in relation to health and the economy. This work also provides further information on health inequalities, eco-nomic and social disparities in the country due to the COVID-19 pandemic and lockdowns and also contributes pragmatic suggestions for overcoming these challenges. These observations will be useful to the relevant local and national officials for improving and adopting novel strategies to face lockdown challenges

## 1. Introduction 

The World Health Organization (WHO) pronounced COVID-19 as a global pandemic on 11 March 2020 when it reported over 118,000 outbreaks, with more than 4000 fatalities in 114 countries around the world [1]. COVID-19 has triggered the world’s largest national lockdowns. Global epidemics are not unusual in the history of human civilisation. Since the Spanish flu pandemic of 1918, the global community has witnessed the impacts of a series of epidemics, such as anthrax in 2001, SARS in 2003, H1N1 in 2009 and Ebola in 2014. The upsurge of coronavirus has become the most serious threat to public health in recent times [2]; it is the fifth pandemic in the last two decades and the ninth in the last century [3]. This virus has virtually brought the world to a standstill. It has made society realise that the world we live in today has no borders for diseases, as the virus has spread to several countries within a very short span of time. COVID-19 is 3 to 30 times deadlier than seasonal influenza in terms of fatality rate [4]. In India, 34,758,481 confirmed cases of COVID-19 infection and 478,325 deaths have been reported as of 22nd December 2021 [5,6]. COVID-19 has serious implications not only for health but for different sectors of the economy due to many industry activities coming to a standstill during lockdowns. The COVID-19 pandemic and the subsequent lockdowns have contributed to significant changes in nearly every area of work and life all over the world [7]. Restrictions of economic activity, including halting production and transactions, have a severe impact on the day-to-day lives of people.

Market failure and externalities are common problems resulting from imbalances in demand and supply of goods and services in the economy. Although wealthier countries have had difficulties revising administrative and regulatory systems, some poorer nations could not quickly respond to the pandemic through closure of their informal economies without risking worse health impacts than those of COVID-19 disease, e.g., starvation [8]. It has been found that without active policies to safeguard or replace the flow of income to vulnerable populations, more than 30 million people may fall into poverty [9]. This review paper addresses the impacts of the COVID-19 pandemic and lockdowns on the health and economy of India. This systematic review provides information on health inequalities, economic and social disparities in the country due to the COVID-19 pandemic and lockdowns and offers pragmatic suggestions to overcome the challenges.

## 2. Methodology

### 2.1. Case Study Area

The review article focuses mainly on the economic and health impacts of COVID-19 and lockdowns in India. Though the government imposed the lockdown to stop the spread of COVID-19 infections, the limitation of people’s movement caused various health difficulties, including mental health disorders, among those living in India’s cities and rural areas. This article uses India as a case study, as the population of this country, particularly those from marginalized groups, suffered serious physical and economic implications as a result of the lockdown, and they faced limitations in accessing healthcare during the lockdown. Those working in unorganized sectors and migrants who previously relocated to urban areas for their livelihood lost their jobs and income and had to return to their hometowns in various parts of India by whatever transportation they could find, including by foot. 

### 2.2. Literature Searches 

A systematic literature review was used for this study. A vast number of studies have been undertaken on COVID-19, but currently, none are based on the latest figures of COVID-19 and lockdowns at a national level for India. Literature was searched using Scopus, Web of Science, and Sciencedirect.com sources. These three databases were selected to ensure the resulting literature was precisely targeted. However, the current study did not use Google Scholar because it was too much work to undertake and filter. Each database search is comprised of relevant search words related to: COVID-19, lockdown and India; additional searches were undertaken for terms including: economic slowdown, health impact, migrants and informal sector. Searching these terms from different sources identified several duplicate papers, particularly those related to COVID-19 in India. As a result of this challenge, a different combination of words was used to search the relevant literature as follows:

1. COVID-19 and India
2. Lockdown and India
3. Lockdown and health impacts in rural India
4. Migrants and greater lockdown

Once the relevant literature was identified by the different search engines, duplicates were removed, and the resultant literature was examined alongside the exclusion and inclusion criteria, which are elaborated in Table 1. This process was done in various stages, where the publication date was originally scanned against the inclusion and exclusion standards, and accordingly, a title and abstract scan was undertaken. Where the title and abstract did not reveal the full scope of a particular paper, the full paper was studied to get the complete information for that particular research article. It was only necessary to undertake this for 120 of the articles. Along with the abovementioned sources, the study also used grey literature and government technical reports. For these types of literature, we used direct government websites, such as the Indian Ministry of Health and Family Welfare and the National Disaster Management Authority, and considered articles related to the search words, then undertook the inclusion and exclusion protocols. 

### 2.3. Study Limitations 

Because of time constraints, the authors did not conduct a literature search using other databases, such as Medline or CINAHL, which would have limited access to some relevant papers. Due to the lack of updated publicly available documents, the authors did not consider the major difficulties of pre-existing health conditions during the pandemic.

## 3. Results and Discussion 

Based on the Web of Science, Scopus, and Science Direct.com, a total of 1205 relevant scientific papers were identified related to the search words. Across these search engines, 432 duplicates were recognised and deleted. Excluding literature not directly related to the search words removed a further 320 articles. This left 453 articles where the titles and abstracts were considered for the initial assessment; as a result of this processes, a further 303 articles were excluded from the evaluation for not meeting the inclusion criteria. At this stage, 150 articles, including 25 full papers, were considered for in-depth analysis. After complete reading of these 150 papers, a further 93 articles were excluded, as they were overly technical in nature. Finally, 57 articles were considered for complete evaluation. Of those, we present the top 10 papers, which are highly relevant and frequently cited papers, in Table 2.

**Figure 1:**
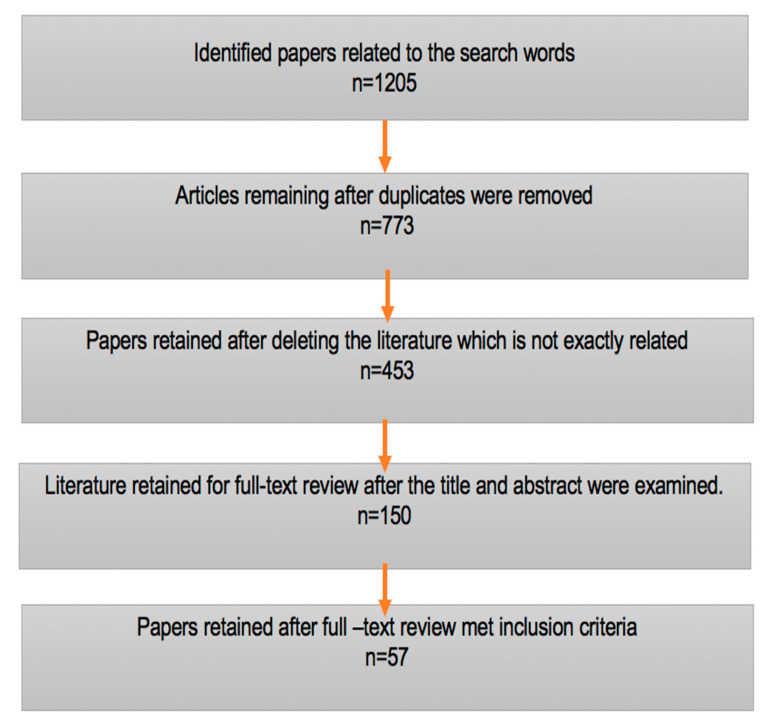
Flow chart of inclusion and exclusion criteria.

Figure 1 also provides information on the systematic exclusion and inclusion criteria of the current study. 

### 3.1. The Great Lockdown and Its Economic Impact 

Inequality and poverty are anticipated to worsen, having a particularly detrimental impact on migrants, casual and informal workers, as well as mental health issues and domestic violence [10]. Considering the larger size of the informal sector, inequality and poverty may worsen in India. COVID-19 lockdowns have had a wide-ranging effect on India’s economic activity. Production, distribution and consumption have been disrupted. Domestic demand has sharply declined, and restrictions on trade and business have been severe. With the continuing decline in demand, extensive supply-chain disruptions will persist for some time, related to the shortage of raw materials. Supply chains are unlikely to normalise for some time still. Several companies are still suffering due to the disruption to China’s supply chains. The further the recession continues, the harder it becomes for businesses to remain stable. In almost all domestic industries, this will have an adverse impact on demand. The potential length of the underlying health crisis remains uncertain at this point. It is challenging to completely understand the magnitude of the disruption presently incurred by the Indian economy [20]. The COVID-19 lockdowns will have implications both at the micro and macro levels for the economy for some time. It will have implications for all sectors, ranging across agriculture, industry and services. 

Though the unlocking process has started in India, the economy has not opened up fully. Transport services have not been fully resumed, which is instrumental to the movement of labour and raw materials. Key sectors of the economy, like construction, infrastructure and manufacturing, have been badly hit. Services like trade, tourism and hotel industries are still not fully operational. A restricted lockdown may continue for some time. The greatest concern, for now, is that a per centage of the population will be forced into extreme poverty or even hunger by the combination of the damage to their livelihoods and disruptions in standard distribution mechanisms [21]. Psychological issues might be particularly difficult for marginalized people or the poorest sectors of society, such as farmers who already face psychological difficulties as a result of previous troubles in the agricultural industry. Every year, almost 16,500 farmers commit suicide as a result of their low socio-economic circumstances, and COVID-19 has the potential to exacerbate this situation [22]. Research by Narayanan (2020) shows that farmers were trapped with their harvests as an Agricultural Product Market Committee (APMC) mandate shut down many states as the initial lockdown was implemented in March 2020, thereby creating food-supply interruption from production to consumption sites [23]. A large population of people depend on the informal sector. The sudden closure of the informal employment has forced unorganised workers to struggle for a daily living. The Asian Development Bank (annual report of 2020) estimated that GDP loss to India is Rs.30.3 lakh crore (in USD $402,722,148,000—four hundred two billion seven hundred twenty-two million one hundred forty-eight thousand dollars (Converted from rupees to USD as per the rate of 18 October 2021—1 USD = Rs. 75.24)), which about 13.7 per cent of GDP. The unorganized workforce and circular migrants who work on informal and temporary contracts are growing in number and are the most insecure and at-risk population [17].

### 3.2. Implications of the Lockdown 

In the event of a health crisis like COVID-19, the pandemic has emphasized the critical necessity to review disaster and public health responses. Despite advancements in the ability of nations and the world to react to public health disasters, the healthcare capability of a number of major countries has indeed been challenged during this pandemic. Even developed countries with sophisticated infrastructure, sanitation and hygiene have been unable to overcome the pandemic’s effects. Lower-income countries, on the other hand, have been overwhelmed, with many countries unable to respond to and manage the pandemic due to a lack of infrastructure and resources, as well as weak administration and poor populations. The pandemic has revealed and exacerbated disparities between low- and middle-income countries, as well as affluent and poorer portions of society. The pandemic has also shown vulnerabilities in worldwide surveillance systems, as well as a failure to detect and limit the outbreak [24]. Lockdown is seen as an important step in slowing the spread of coronavirus globally [25]. Many countries are currently in some level of lockdown to slowdown the spread of the virus. India’s prime minister announced a nationwide three-week lockdown from midnight on 25 March 2020 to 14 April 2020, stating that it was an inevitable and successful step to break the COVID-19 infection cycle. After that time period, a process of phased unlocking started, with certain relaxations permitted. In India, the lockdown continues to exist, with certain relaxations as a preventative measure for reducing the spread of the virus.

Lockdown has had mixed impacts on health and the economy. Social distance is an effective way of breaking the infection cycle, and lockdown plays a vital role in that. Restrictions on movement of people and public gatherings, as well as the shutdown of economic activities, help reduce the spread of infection and restrict the number of positive cases and COVID-19 vulnerability. There seems to be a substantial reduction in the growth of cases because of lockdown [14]. Conversely, lockdown has also forced individuals to stay at home, which has affected their physical, mental and social health. Schools and universities remain shut, disrupting students’ education. Those who are unable to access the Internet to study online or who do not have access to a computer are more likely to suffer educationally. Those who are about to enter the workforce face difficulties due to shifting corporate needs and fewer job openings. All of these elements contribute to mental stress, and the implications of this pandemic could be severe in cases where people are already suffering from mental health issues [10]. 

Children of parents who are infected by COVID-19 or who work in health services are particularly vulnerable [26]. The pandemic situation has changed the workplace atmosphere drastically, leading to increased work and unfavourable and stressful interactions between healthcare professionals. When attempting to manage living as a healthcare practitioner and as a family member, working with highly infectious clients has resulted in culpability for subjecting family members to infection [16]. The global spread of coronavirus has also contributed to many mental health issues and a decline in general well-being [12]. The lockdown has had substantial implications for economic activities and the economy. The shutdown of industries and restrictions on the mobility of labour has created problems for economic growth, employment and distortions in demand and supply.

### 3.3. Migrant Workers

Due to preventative measures implemented to arrest the disease’s progression, 81 per cent of all workforces are impacted by partial and full lockdowns around the world. Employees suffered a decline in pay and fewer work prospects as businesses and firms struggled financially and collapsed [27]. However, the worst effects have been on informal workers, who have been least safeguarded among all groups of labour classes [27]. A total of 90 per cent of Indian employees are employed in the unorganized sector, with untold millions working in cities far from their traditional, typically rural homes. When the Indian government declared an immediate ‘lockdown’ in March 2020 to contain the pandemic, migrant informal workers were trapped in a struggle for economic survival as a result of job losses, starvation, and persecution by state containment officials and frightened populations maintaining ‘social distance’ [17]. When notification of the shutdown declaration was announced, many migrant employees were immediately put out of work. Panic-stricken workers gathered in huge numbers at bus stations and highways, expecting a quick return to their distant, rural villages. The purpose of the lockdown step was to implement ‘social distancing’ to reduce the spread of infection, yet there was no option for migrant workers in their temporary, crowded urban homes, as they had to experience those conditions without jobs, income or social security. Reaction to the directive on preventive confinement over the following few days and weeks revealed the vulnerability, confusion and precariousness of migrant workers’ lives and situations. Currently, 400 million people in India’s informal sector, accounting for 90 per cent of the working population, are at risk of falling into poverty. Angered by migrant workers’ plight, public policy academics have described the COVID-19 quarantine as a “choice between virus and starvation” [28]. ‘Scheduled tribes’ are classified as among India’s poorest populations, often moving seasonally for employment as farm labourers. When India entered its phase-3 lockdown from the first week of May 2020, tales of horrific tribulations and precarious travels emerged, with migrant labourers walking, cycling and even forced to migrate on concrete mixer trucks with their children [29]. 

#### 3.3.1. Road Traffic Accidents 

At least 1461 accidents occurred during the nationwide lockdown, which lasted from 25 March 2020 to 31 May 2020. In total, there were 750 people who died, with 198 of them being migrant workers. There were 1390 persons who were injured in total. A total of 26.4 per cent of the overall deaths during the lockdown happened as a result of people attempting to return home, and 5.3 per cent of them were deaths of essential service providers. Other road users were involved in 68.3 per cent of all fatalities [30]. Therefore, it is imperative to examine why migrant workers had to undergo this ordeal. 

#### 3.3.2. Lockdown and Travel Home

With the subsequent announcement of the lockdown’s extension, many people experienced joblessness, followed by homelessness, as their savings ran out [31]. Their situations and their hopelessness worsened. It is crucial to remember that ‘migrants’ are not a monolithic group; they work in both the formal and informal sectors and at all levels of the occupational ladder. Following the closure imposed by COVID-19, both international and domestic migrants yearned to return home [32,33]. The Indian government announced the ‘Vande Bharat Mission’ on 7 May 2020, which had the goal of reconnecting Indians who were stranded in other countries due to pandemic-related restrictions. The Indian government returned 12,000 Indians from Gulf countries and another 15,000 from the United States, the United Kingdom, the Philippines, Bangladesh, Malaysia and the Maldives as part of Phase I of the Vande Bharat Mission [34]. Phase II began on 16 May, and between 16 and 22 May 2020, around 32,000 Indians from 21 countries were brought back. India used private airlines to evacuate individuals during the Mission’s second phase. The majority of the 45,000 Indians who had returned by the end of May 2020 were migrant labourers (8069), but this group also included students (7656) and professionals (5107). Air India, the government carrier, took the lead in reuniting stranded passengers from more than 60 nations. Moreover, over 5000 Indians returned to India via immigration checks in Nepal and Bangladesh [35]. 

### 3.4. Health Implications

The COVID-19 pandemic is the biggest challenge for the health sector and health policy planners. It has direct and indirect implications for the health and well-being of people. People are directly affected by the spread of infection and hospitalisation, which can lead to loss of work for hospital attenders in the family. One infection in a family can lead to infection of multiple family members, particularly in cramped living conditions.

1. Sudden job losses have resulted in the depletion of income and severe difficulties for millions of urban and rural populations, most of whom work without contracts in the informal sector [36]. As a consequence, several million informal workers and their families have experienced poverty, hunger and acute undernourishment.
2. Domestic conflict, violence and depression have also increased [37].
3. Health inequalities have exposed vulnerable groups to more risk. It is time to recognise the contribution of the informal sector to the national economy. Lockdown has shattered the mental health of a large population and has increased chances of exposure to a disturbed daily routine, which results in reduced human happiness and well-being [15].
4. The approaches to address COVID-19 and management of COVID 19 may generate inequalities, as well as problems in health services, interrupt essential regular operations and require redistribution of existing healthcare workers across health systems. The impact of the pandemic also has consequences for routine non-COVID-19 health services. In low-income contexts, this has meant people spending excessive amounts of time waiting for, e.g., antenatal treatment, contraceptive counselling or reproductive health facilities in crowded hospitals, which further raises the risk of spreading infection.
5. Fear and stress result in a higher risk to individuals of experiencing both physical and mental health issues [11].
6. Misinformation related to COVID-19 has been spread partly due to fear, stigma and anxieties [38].
7. People suspected of having COVID-19 infections experience an increased level of stigma [39].
8. Rising levels of violence against help/care professionals is also a big concern, though they risk their lives to serve society in this difficult pandemic situation [40,41].
9. The ICMR stated that India tests 24 people per one positive, while Japan tests 11 people for one positive, Italy tests 24 people for seven positives and the USA tests 24 people for 5.3 positives; in comparison with other countries, there have been fewer COVID-19 cases per test in India [42].
10. During the pandemic, one out of every ten women in India polled needed domestic abuse services.
11. Mid-day meals were stopped in schools, which has disproportionately affected poorer children’s health, as well as malnutrition among children [43].

In India, there were about 927,606 severely acute malnourished children aged six months to six years [44]. Malnutrition has been the cause of about 68 per cent of deaths of children under the age of five years [45]. According to the Global Hunger Index of 2021, India ranks 101st out of 116 countries, with a score of 27.5 (level of serious) [46]. Earlier in 2020, India ranked 94th, and in 2021, it has fallen further [46] There is a lack of access to health infrastructure/resources for needy people [47].

### 3.5. What Is Special in the ‘Shramik Special’? 

The government took cognisance of the problem encountered by migrant workers in the informal sector in cities in the wake of the COVID-19-induced lockdown. It announced 800 trains, paradoxically named ‘Shramik Special’. They started to operate on 1 May 2020 and ferried nearly 10 lakh migrant workers trapped in various parts of the country. Uttar Pradesh and Bihar received the maximum number of workers through these trains, while Gujarat and Kerala were the largest ‘senders’ of workers. The swelling load of workers wanting to return home led to an increase in the number of passengers a train could carry. It was increased from 1200 to 1700. This also resulted in a maximum of three intermittent stops on each route starting 11 May 2020, thereby incurring a cost of about INR 800 lakhs per service [48]. This cost was mandated to be shared by the centre and the state in a ratio of 85:15. This benevolence of the government had a rider that the train would ply only if the occupancy was 90 per cent, overlooking the humanitarian requirement of dismissing the economic benefits. However, 100 ‘special’ trains were scheduled to operate daily to ease the travel requirements of the stranded workers [49]. 

As the trains were started, procuring tickets and travel permission was unorganised, and the cost was arbitrarily exorbitant. This impacted the psychosocial well-being of the workers in need of travel. Those stranded offshore could register for travel back. They were updated about the current situation of travel possibilities, as well as the COVID-19 situation. Workers stranded within the country in different cities had to go through a cumbersome and unfriendly process. Each district had its own stipulations. Workers were unable to fill the mandatory online forms for procuring the tickets for a variety of reasons. From a lack of awareness, reading and writing skills and barriers in the use of the online portal to the disgust and embarrassment of running from one office to the other and financial concerns, the restraints were too many to be overcome—all this for buying a train ticket to reach home. If instead of ‘special trains’, regular trains continued to ply with limited capacities, travel is likely to have been smoother than the chaos created by special trains. The process of procuring tickets translated into a means to extract money from already distressed workers. The reckless process of getting tickets forced some to start walking [48,49].

It seemed essential to examine the need for special trains when we already have one of the densest networks of rails. The existing network—albeit with lower occupancy—while plying on existing lines and stoppages, would have saved travellers a lot of dis-order created during the plying of special trains. The trains would not have ‘lost their way’ on the tracks and reached places other than the destination nor seen long delays, death and disease among travellers. Shortages of water and food, unclean toilets and zero adherence to disease distancing and sanitisation became the norm on the special trains. The special trains functioned more poorly than the ordinary ones, leaving behind a horde of gruelling stories of pain and discomfort.

## 4. Policy Implications for Migrant Workers

For the first time, the COVID-19 crisis has placed migrant workers’ problems at centre stage for social protection policy concerns. The migrant worker tragedies that occurred as a result of the COVID-19 lockdown highlight the importance of consolidating social policy measures. Within debates about growth and social security, migrant workers hold a special place. They have helped propel the global economic engine, but they have not benefited greatly from the process themselves. In the aftermath of COVID-19, they are turned against by the same factors that make them appealing as a labour force [17]. The ongoing crisis of poor migrants masks the bigger story: mass unemployment and vulnerable, unregulated jobs marked by post-COVID-19 work environments. These should provide better security and fairer job conditions for informal workers based on the existing policy framework. States also need to address the challenges of rural workers facing adverse circumstances, affecting the livelihood of millions. 

Rural social security programs should be extended to urban areas, including public works under MGNREGA (the Mahatma Gandhi National Rural Employment Guarantee Act, 2005) and other welfare initiatives, such as child and maternal protection and the public distribution system, should be made accessible. Criteria of the PDS need to be relaxed in this context to accommodate informal workers who have lost their jobs. Farmers and the agricultural rural sector also require government assistance as informal urban labour and migrant remittances dry up [17]. The Food Corporation of India has a buffer stock of excess food grains due to be distributed to migrant workers. The total stock was 78 million tonnes (171,960,564,504 pounds—one hundred seventy-one billion nine hundred sixty million five hundred sixty-four thousand five hundred four pounds), but what was actually needed was 21 million tonnes (46,297,075,059 pounds—forty-six billion two hundred ninety-seven million seventy-five thousand fifty-nine pounds); the surplus could be distributed to the poor. Food security has to be ensured for the entire population in the midst of this crisis. 

## 5. Policy Implications and the Critical Role of the Public Sector

The pandemic had a huge economic impact on international trade, interest rates, financial sector liquidity and market dynamic shocks, among other things. It is uncertain when or how long it will take for the world’s economies will recover from the pandemic. A global economic crisis as a result of the COVID-19 pandemic appears to be unavoidable, but the length of the recession is unknown. Governments, policy makers, healthcare professionals and the general public will all need to work together to complete recovery operations.

1. Individuals should have easy access to critical pandemic-fighting goods, like face masks and hand sanitizer, and adequate testing facilities should be provided to the public [50].
2. The COVID-19 pandemic complicates the existing healthcare service delivery system. Interdisciplinary research and scientific investigation are necessary to understand the situation. To minimize the pandemic impact, the neglected tropical diseases community, particularly in low- and middle-income countries, must respond quickly, wisely and collaboratively with decision makers and key stakeholders across sectors [13].
3. To address the pandemic situation, which necessitates new and innovative responses from stakeholders, multi-stakeholder participation in pandemic management must be investigated. Multi-stakeholder and spatial decision support systems have proven to be an effective model for identifying potential pandemic sources and regulating worldwide spread. To address COVID-19, transdisciplinary approaches to structuring and decision making tend to be very helpful. Furthermore, conceptual multi-stakeholder and spatial decision support systems can help enrich crisis-management decision making by bringing out a synergic link amongst multi-stakeholders [19].
4. Increasing capacity for income generation, improving working conditions for labour and easy access to public services are required. Tailored packages for vulnerable populations (informal sector, small and micro-entrepreneurs, women, indigenous populations) have to be created to support them.
5. Empirical analysis suggests that proper management of reverse migrants and an increased role of people’s participation in adhering to social distancing are important for restricting COVID 19 cases [18].
6. The government has a crucial role in encouraging and facilitating participation of the public. With the unlocking process in force, migrant workers will again try to move to seek jobs. The government needs to ensure they have databases of labour movements and safety and social distancing measures in the workplace.
7. The concept of health has to be expanded beyond biomedicine and hospital costs to reflect the direct and indirect cost of health impacts.
8. The pandemic challenges of the international community have to be handled with global cooperation and security.
9. Investment in emergency preparedness is needed to manage the crisis. Coordinated funding in public health emergencies can improve access to measurement, prevention, treatment and control of the virus.
10. Funding the resumption of economic activity requires financial supports, such as by providing easy access to credit through the Credit Guarantee Fund Trust Scheme to SMES (small and medium enterprises).
11. The government and the Reserve Bank of India (RBI) have taken a number of steps, including a moratorium loan forgiveness regulatory forbearance, updated NPA (non-performing asset) laws and easing the credit cycle, but these steps must be implemented properly.
12. If necessary, measures are not taken to manage the crisis, it can lead to a national emergency. Linkages between all levels of state and non-state actors are critical for ensuring the population’s health and safety. The need for early public health countermeasures and mitigation of the spread of the virus has highlighted the appropriateness of support by state, local and other health departments.

Innovative infection-control methods can lead to a more effective healthcare service delivery system [51]. Reinforcing self–discipline among citizens can reduce the spread of the virus [52]. The focus on universal health coverage can result in better regulated public healthcare. It is the appropriate time to accept the importance of public health surveillance. There is an urgent need for coordinated efforts in the preparedness for and response to public health emergencies. Greater engagement by multi-stakeholders and participation of citizens would facilitate better identification of needs and priorities. Transdisciplinary approaches are necessary to create a database for evidence-based health policies to prioritise areas of health. Global economic well-being and emphasis on equal opportunities would improve health governance. 

The government and the Reserve Bank of India have adopted and declared several fiscal and monetary policy actions, but notable economists believe that the government has to spend more, regardless of the GDP numbers and budget deficit. In reality, more attention should be paid to the most vulnerable members of society and sectors, particularly micro, small and medium enterprises and the non-essential commodities sector, which are the hardest hit by the pandemic’s demand contraction. The pandemic is requiring unique, comprehensive and inventive measures [10]. National governments and states have initiated many measures, and their role is also important. For example, in Tamil Nadu, considering the rise of cases, the government has scaled up infrastructure and testing centres, and the recovery rate in Tamil Nadu is now more encouraging. The need for greater involvement of central banks and state governments and promotion of national and international community participation to improve services of the health sector to deliver high-quality care to the public is crucial. Economic restructuring, like debt relief and social welfare flagship programmes, are needed to deal with the current pandemic situation. To promote multilateral cooperation, a proactive and coordinated effort is needed at this juncture. Panchayats (village councils) and local governments have to play a significant role. Local governments need to be empowered and involved in drawing up plans and supporting economic activity, livelihood programmes, development of rural infrastructure and social welfare schemes, starting at a grassroots level. This will help with immediate employment needs and longer-term developments. It has been argued that the state and central governments should provide tax holidays for the MSME sector for five years, with more incentives and subsidies to be provided. An export-promotion and import-substitution measure should be taken. To compete with the international market, central governments should provide infrastructural facilities to MSMEs. Active policies to protect informal workers in the country are required.

## 6. Scope for Future Research

There is a need to conduct impact-assessment studies connected to widening inequities between affluent and poor due to the pandemic by applying a transdisciplinary approach involving multi-stakeholder participation. It is also important to research the health and well-being of frontline health workers during the pandemic, as their workload is overwhelmed.

## 7. Conclusions

The COVID-19 pandemic and subsequent nationwide lockdowns in India have had severe implications for health, the economy and employment. To evaluate the economic and social effect in the fullest sense, there is a need for a holistic approach that includes an assessment of the increased burden of poverty, hunger and mental health, rather than only looking at COVID-19 cases and related fatalities. Increases in emergency spending on public health infrastructure and revamping of administrative and regulatory frameworks would significantly improve public healthcare. A systematic and scientific approach to unlocking is needed at the right time, and comprehensive measures are required for safe and decent workplaces and addressing the issues of the informal sector. Multi-stakeholder participation and systematic efforts by national and state governments, including local governments, are crucial for ensuring social distancing, enhancing healthcare management of COVID-19 and resuming economic activities. Measures are needed to ensure resumption and the opening up of economic activity and to restore supply chains to provide income and employment to people. To protect the income of people, some focused steps must be taken as measures for safeguarding livelihood and food security. Increasing budgetary allocations for social services and the implementation of an additional wide range of social assistance programmes would facilitate a healthier life for the crisis-affected population.

More social protection measures for migrant workers are needed, as they face challenges when measures like lockdowns are implemented. The state must establish a system for regulating the number of working hours and wages for migrant workers. During lockdown, the state must provide direct financial transfers to poor urban and rural people to prevent hunger, poverty, and malnutrition. Alternative livelihood alternatives for economically disadvantaged persons must be identified and implemented in order to improve their purchasing power for essential commodities. People employed under the MGNREGA (the Mahatma Gandhi National Rural Employment Guarantee Act 2005) in India, for example, should be allowed to work for an increased number of days, and their wages should be increased to supplement their income. Because rural areas lack testing facilities, the government should establish a nationwide mobile COVID test laboratory focusing on rural communities. People who travel overseas regularly should be subjected to tighter restrictions, as they may transmit and disseminate novel virus strains from one country to another. Frontline workers and other stakeholders involved in preventing virus spread require capacity-building programmes since they bear a significant amount of responsibility for educating people about preventive measures and treating persons who have infections. When the virus spreads rapidly, a support structure must be put in place to look after the mental health of frontline workers who work long hours without much respite. The government should use multi-stakeholder input to develop evidence-based public health strategies to prevent the spread of infections. Data science, technology, and epidemiological perspectives can all work together to anticipate the spread of a virus in the future, allowing public health actions and education to be carefully framed to protect people from infection. Strengthening and establishing a solid public health infrastructure, including human resources for health, will require strong political will to handle pandemic situations.

## Figures and Tables

**Table 1:** Inclusion and Exclusion criteria.

Number	Inclusion Criteria	Exclusion Criteria
1	Articles published in between 2019–2021	Articles published before 2019
2	Articles published on COVID-19, lockdown, health impacts and improvement strategies, India	Articles that are relevant but too technical (mathematics and lab results), such as vaccine development
3	Articles directly or indirectly related to the search words	Articles not very relevant to the search words
4	Articles with new results, which should be applicable to any geographical area	Articles that are complicated to understand, with incomplete or biased results
5	Other pandemics related to the COVID-19 pandemic	Overly technical papers or papers on unrelated pandemics

**Table 2:** Top 10 papers of the systematic review.

No	Source	Article Title	Article Type	Article Information
1	[10]	“An assessment of socioeconomic impact of COVID-19 pandemic in India.”	Review	The COVID-19 epidemic has caused tremendous losses worldwide, but India, as an emerging country, is likely to be disproportionately affected in every industry. The service sector, which is the main driver of financial development and the biggest contributor to GDP, has been severely harmed as a result of various limitations on mobility, such as the temporary suspension of tourism and hospitality, the limited availability of transportation, the closure of schools and colleges, etc. The total economic and sectoral losses are determined by the intensity and duration of the crisis. In addition to economic damage, the societal impact of this coronavirus outbreak and unparalleled crisis is harsh, with substantial social and psychological issues.
2	[11]	“A study on impact of COVID-19 lockdown on psychological health, economy and social life of people in Kashmir.”	Original research	COVID-19 lockdown has an adverse impact on the mental health of different categories of people, particularly public, casual employees, students and health workers.
3	[12]	“The psychological impact of quarantine and how to reduce it: rapid review of the evidence.”	Review	The global spread of COVID-19 has also contributed to many mental health issues and a decline in general well-being.
4	[13]	“Efforts to mitigate the economic impact of the COVID-19 pandemic: potential entry points for neglected tropical diseases.”	Review	The COVID-19 pandemic complicates existing healthcare service delivery systems. To minimize the pandemic impact, the neglected tropical diseases community, particularly in low- and middle-income countries, must respond quickly, wisely and collaboratively with decision-makers and key stakeholders across sectors.
5	[14]	“The positive impact of lockdown in Wuhan on containing the COVID-19 outbreak in China.”	Original research	Restrictions on movement of people and public gatherings and shutdown of economic activities help stop spreading infections and restrict the number of positive cases and COVID-19 vulnerability. As a result of the lockdown, there seems to be a substantial reduction in cases.
6	[15]	“Job loss and mental health during the COVID-19 lockdown: Evidence from South Africa.”	Original research	Health inequalities have required vulnerable groups to live more riskily. Recognising the informal sector’s contribution to the national economy, lockdown has shattered the mental health of a large part of the population and has increased chances of exposure to disrupted daily routines, which results in reducing human happiness and well-being.
7	[16]	“Social stigma during COVID-19 and its impact on HCWs outcomes.”	Original research	The pandemic situation has changed the workplace atmosphere drastically, leading to increased work hours and unfavourable and stressful interactions for healthcare professionals. When required to manage living as a healthcare practitioner and as a family member, working with highly infectious clients has subjected family members to infection.
8	[17]	“Social policy, COVID-19 and impoverished migrants: challenges and prospects in locked down India.”	Review	Unorganized workforce and circular migrants who work on informal and temporary contracts are growing in numbers as the most insecure (socio-economic) and at considerable community risk. Migrant informal workers face difficulties, including job losses, starvation and persecution by state containment officials.
9	[18]	“Socioeconomic determinants of COVID-19 in Asian countries: An empirical analysis.”	Original research	The proper management of reverse migrants (returning back from urban places to native (rural) places) and an increased role of people’s participation in adhering to social distancing are important for restricting COVID-19. Government is crucial to successful participation by society. When lockdowns ease, migrant workers again move to seek jobs. The government needs to have databases of labour movements and ensure safety and social distancing measures in the workplace.
10	[19]	“Multistakeholder Participation in Disaster Management—The Case of the COVID-19 Pandemic.”	Review	The combination of multi-stakeholder and spatial decision support systems has shown to be the most effective model for identifying probable pandemic sources and controlling global spread. Multi-stakeholder and spatial decision support systems can help enable crisis-management decision-making by bringing out a synergic link amongst multi-stakeholders.

## Data Availability

Data are available on request from the authors.
